# Blackwater fever in an uncomplicated *Plasmodium falciparum* patient treated with dihydroartemisinin-piperaquine

**DOI:** 10.1186/1475-2875-13-96

**Published:** 2014-03-14

**Authors:** Chanthap Lon, Michele Spring, Somethy Sok, Soklyda Chann, Rathvichet Bun, Mali Ittiverakul, Nillawan Buathong, Khengheng Thay, Nareth Kong, Yom You, Worachet Kuntawunginn, Charlotte A Lanteri, David L Saunders

**Affiliations:** 1 Armed Forces Research Institute of Medical Sciences (USAMC-AFRIMS), Phnom Penh, Cambodia; 2 Department of Immunology and Medicine, US Army Medical Corps, Armed Forces Research Institute of Medical Sciences (USAMC-AFRIMS), Bangkok, Thailand; 3 Royal Cambodian Armed Forces, Phnom Penh, Cambodia; 4 The National Center for Parasitology, Entomology and Malaria Control (CNM), Ministry of Health, Phnom Penh, Cambodia

**Keywords:** Malaria, Plasmodium falciparum, Cambodia, Blackwater fever, Dihydroartemisinin-piperaquine

## Abstract

The mechanism of massive intravascular haemolysis occurring during the treatment of malaria infection resulting in haemoglobinuria, commonly known as blackwater fever (BWF), remains unknown. BWF is most often seen in those with severe malaria treated with amino-alcohol drugs, including quinine, mefloquine and halofantrine. The potential for drugs containing artemisinins, chloroquine or piperaquine to cause oxidant haemolysis is believed to be much lower, particularly during treatment of uncomplicated malaria. Here is an unusual case of BWF, which developed on day 2 of treatment for uncomplicated *Plasmodium falciparum* infection with dihydroartemisinin-piperaquine (DHA-PIP) with documented evidence of concomitant seropositivity for Chikungunya infection.

## Background

Haemoglobinuria is a feature of complicated malaria syndromes as defined by the World Health Organization (WHO) [1]. Blackwater fever (BWF) is due to massive haemolysis of red blood cells in the blood stream with subsequent haemoglobinuria, anaemia, often accompanied by renal failure. By published reports, BWF occurs most often in *Plasmodium falciparum* infections, but has also been documented in *Plasmodium vivax*, *Plasmodium malariae* and mixed infections [2-6]. Though the exact mechanisms involved remain unclear, several factors appear associated with BWF such as G6PD deficiency, severe malaria infection, treatment of malaria with amino-alcohol drugs, particularly quinine, and presence of other viral or bacterial infections [3,4,7].

## Case presentation

A 43-year old male Cambodian farmer was screened and enrolled on 21 July 2013 to participate in a clinical trial evaluating the efficacy of a course of dihydroartemisinin-piperaquine (360 mg/2880 mg) divided over three days for uncomplicated *P. falciparum* malaria with or without a single dose of primaquine 45 mg to prevent transmission of gametocytes (NCT 01849640). During the three days before enrollment, he reported experiencing mild fever, chills and fatigue, and self-treated with paracetamol and ampicillin. On the day of enrollment, he was found to have *P. falciparum* infection with 31,218 parasites per μl on peripheral blood smear at an outside medical facility, and was referred to the study team. He denied having taken any anti-malarial or traditional herbal medications, but he did inform the study team that he has been consuming 1–2 cans per day of an energy drink containing taurine, caffeine, and B vitamins for the past year. He reported no chronic underlying illnesses. He had suffered four malaria episodes since 2010 which were variously treated with chloroquine, artesunate and mefloquine, but reported no prior history of adverse reactions to anti-malarials or history of BWF.

Initial vital signs included a heart rate of 70, blood pressure of 108/69 mmHg, and a tympanic temperature of 38.1°C. He was not ill-appearing and had no jaundice, icterus, conjunctival pallor or rash. The remainder of the physical examination was unremarkable, including a normal neurologic examination and no hepatosplenomegaly. Urine was light tea coloured. Initial laboratory assessment revealed mild anaemia (Hb, 10.3 g/dl and HCT, 30.3%), low serum calcium (6.7 mg/dl), and normal serum creatinine (0.92 mg/dL). His G6PD activity was normal (9.8 U/g Hb, normal range 4.6-13.5 U/g Hb), and serial 10-second surface EKG tracings were normal, with an average manual QTcF of 444 ms.

Twenty-eight hours following admission, after completing the first two doses of DHA-PIP, he was observed to have dark 'coca-cola’ coloured urine. His fever was still present and new signs and symptoms developed including pallor, dizziness, fatigue, anorexia and semi-watery diarrhea with abdominal pain in the right lower quadrant. Acute renal injury with an increase in serum creatinine from 0.92 to 1.7 g/dL, along with a decline in haemoglobin to 8.1 g/dL (21% decrease from baseline), was observed. He was switched to intravenous artemether (160 mg on day 1, 80 mg per day on days 2–5) for severe malaria with blackwater fever. He continued to have an appropriate response to anti-malarial therapy, with a decline in peripheral parasitaemia to 286 parasites/μl within 48 hours, and was smear negative by day 5 (Table 1). Primaquine therapy was withheld.

**Table 1:** Key laboratory values

**Parameters (normal values)**	**D1**	**D2**	**D3**	**D5**	**D7**	**D9**	**D10**	**W2**	**W3**	**W4**	**W5**
**WBC** 10^3^/μL (4.0-10.0)	3.0	4.3	4.54	5.49	6.5	7.3	**-**	6.1	**-**	5.3	5.3
**Blood smear** (parasites/μl)	31,218	4,050	286	Neg.	**-**	**-**	**-**	**-**	-	Neg	Neg
**HB** g/dL (13–18)	10.3	8.1	8.4	7.9	8.8	8.5	-	7.7	-	8.3	8.2
**PLT** 10^3^/μL (140–450)	71	68	70	113	215	283	-	347	-	264	193
**Cr.** mg/dL (0.8-1.3)	0.92	1.7	1.8	2.71	2.90	3.79	3.29	2.9	2.21	1.8	-
**BUN** mg/dL (7–18)	20.5	25.4	27.85	36.91	23.74	23.0	19.7	19.8	20.2	16.2	-
**AST** IU/dL (15–37)	43.6	261	249	265	111	58.0	-	36.7	-	-	-
**Tot. bil.** mg/dL (0–1.0)	0.818	2.95	2.2	2.1	0.809	0.595	-	0.5	-	-	-
**Dir. bil.** mg/dL (0.0-0.5)	-	-	0.2	0.3	-	-	-	-	-	-	-
**K +** mEq/L (3.5-5.1)	3.68	3.93	3.7	3.8	3.95	4.34	4.29	4.1	3.74	3.39	-
**Na +** mEq/L (136–148)			130	142							
**CHIKV (IgM)**			pos.								

WBC; white blood cell (10^3^/μL). HB; haemoglobin (g/dL). PLT; platelet (10^3^/μ), Cr; serum creatinine (mg/dL). BUN; blood urea nitrogen (mg/dL). AST; aspartate amino transferase (IU/dL). Tot. bil.; serum total bilirubin (mg/dL). Dir.bil.; serum direct bilirubin (mg/dL). K+; serum potassium (mEq/L). Na+; serum sodium (mEq/L). CHIKV (IgM); Chikungunya virus (Immunoglobulin M). pos; positive.

On day 3, he continued to experience fatigue, anorexia and jaundice. Serum creatinine continued to rise to a maximum 3.79 g/dL on D9 although there were never any electrolyte abnormalities. Other laboratory abnormalities included maximum indirect hyperbilirubinaemia of 2.95 mg/dL, aspartate aminotransferase of 265 U/L, and alkaline phosphatase of 163 U/L peaking on days 2, 5 and 7 respectively. A single unit blood transfusion was given on day 4 for a continued low haemoglobin level (8.4 g/dL) with levels thereafter remaining stable at approximately 8.0 g/dL (7.7-8.8 g/dL). Serology for co-infections was negative for IgM against leptospira, all four dengue serotypes, scrub typhus, HIV and *Salmonella typhi*, but positive for reactive IgM against chikungunya virus. Urine colour normalized on day 7, and serum creatinine returned to 1.8 mg/dL within 4 weeks, and there was normal to high urine output throughout the admission.

## Discussion

There are not well-defined predictive risk factors for BWF, and the syndrome is often diagnosed only after malaria patients undergoing treatment are noted to have dark-coloured urine. The mechanisms of BWF remain unknown though it has been associated with low levels of *P. falciparum* parasitaemia, partial malaria immunity, G6PD deficiency, and treatment with amino-alcohol drugs [2-5,7]. In this case, the patient had no risk factors above for developing BWF and had been treated uneventfully for malaria on four prior occasions with various drugs including mefloquine over the past three years. A history of multiple malaria infections are associated with BWF, and this, along with a likely concomitant partial immunity, seems to have been his only risk factor [2,4,5,8]. The patient seemed to have a mild case of BWF as he had stable vital signs, few signs/symptoms, and electrolytes within normal limits suggesting preserved renal function despite acute injury. This is similar to a recent prospective case series in nearby Vietnam, which reported overall mild severity and better outcomes [1,2], but in contrast to previous reports of high mortality with a 26% case fatality rates in European patients [9-11] and 23% in African patients [12] with more severe malaria.

This is the first report of a patient with uncomplicated malaria who developed BWF after administration of DHA-PIP. BWF is classically associated with the amino-alcohols quinine, mefloquine and halofantrine of which quinine is the most common culprit. Since chloroquine was introduced after World War II supplanting quinine as first-line treatment of uncomplicated malaria, there has been an observed decrease in BWF [1]. A recent report suggested that metabolism of quinine by the cytochrome P450 3A4 enzyme may be responsible for increasing oxidative stress within erythrocytes, making these cells more vulnerable to haemolysis in those with malaria and/or G6PD deficiency [13]. The role of artemisinin compounds in such potential oxidant acute haemolysis remains poorly understood [14]. Parenteral artesunate was associated with low, but comparable, rates of BWF compared to quinine in a large trial of paediatric patients with severe malaria (0.7% *versus* 1.2% respectively) [6]. Dihydroartemisinin-piperaquine (DHA-PIP) is a combination of a potent, rapid acting artemisinin derivative, combined with a long-acting 4-aminoquinoline (bis-quinoline), similar to chloroquine. Neither chloroquine, nor its more recently introduced analogue piperaquine, have been associated with blackwater fever [1,15]. There has been one published report of a patient who developed BWF three weeks after stopping prophylactic chloroquine and proguanil [3]. The dihydroartemisinin component contains a highly active endoperoxide bridge and like artesunate, may also be capable of inducing oxidant haemolysis, but has not yet been reported to be a cause of blackwater fever [14]. The patient reported consuming daily doses of taurine, which is involved in many crucial physiological processes [16]. However, its role is not clearly understood and the influence of high taurine doses is uncertain. There has so far been no evidence to implicate long-term ingestion of taurine as a risk factor for haemolysis, though synthetic taurine is obtained from isoethionic acid (2-hydroxyethanesulfonic acid), an oxidant haemolytic.

In this case, the patient did have serologic evidence of Chikungunya infection, but he lacked classic findings of Chikungunya including arthritis, arthralgia, petechiae or maculopapular rash. In a 2001 study by Bruneel *et al*. [5] five of eight BWF patients were found to be co-infected with viral infections including hepatitis C, B and Epstein-Barr virus. However, these co-infection have yet to be proven as significant factors influencing the haemolytic potential of BWF [17]. The strong temporal association here of administration of DHA-piperaquine and onset of haemolysis and haemoglobinuria suggests that clinicians treating uncomplicated malaria patients with DHA-piperaquine, and likely other combinations containing artemisinin derivatives, should be aware of the rare possibility for development of blackwater fever during or shortly after therapy.

## Consent

Ethical approval and patient informed consent was obtained from the patient for the study and for publication of this case report.

## Competing interests

The authors have declared that there are no financial or non-financial competing interests.

## Authors’ contributions

CL, MS, SS, RB, SC, CL1 and DS designed and wrote the article. CL, MS, CL1, DS, MI, NB conducted the study. SS, RB, SC and CL diagnosed and treated the patient and collected all the clinical and diagnostic laboratory data. KT, NK, YY, WK and laboratory team designed, performed and assessed the laboratory tests. All authors read and approved the final version.
